# Effects of titanium oxide coating on the antimicrobial properties, surface characteristics, and cytotoxicity of orthodontic brackets - A systematic review and meta analysis of in-vitro studies

**DOI:** 10.1016/j.jobcr.2023.05.014

**Published:** 2023-06-24

**Authors:** Lichi Ashwin Solanki, S.P. Saravana Dinesh, Ravindra Kumar Jain, Arthi Balasubramaniam

**Affiliations:** a Department of Orthodontics and Dentofacial Orthopaedics, Saveetha Dental College and Hospital, Saveetha Institute of Medical and Technical Sciences, Saveetha University, 162, Poonamallee High Road, Chennai, 600077, Tamil Nadu, India; b Department of Public Health Dentistry, Saveetha Dental College and Hospital, Saveetha Institute of Medical and Technical Sciences, Saveetha University, 162, Poonamallee High Road, Chennai, 600077, Tamil Nadu, India

**Keywords:** Antimicrobial activity, Bracket, Microbiology, SEM, Titanium oxide

## Abstract

**Objective:**

The objective of this review is to systematically analyze the available literature on the effects of titanium oxide (TiO₂) coating on the antimicrobial properties, surface characteristics, and cytotoxicity of orthodontic brackets.

**Methods:**

In-vitro studies reporting on the effects of Titanium oxide (TiO₂) coatings on antimicrobial properties, surface roughness, cytotoxic activity and bacterial adhesion of orthodontic brackets were included in the review. Electronic databases such as PubMed, SCOPUS, Web of Science and Google Scholar, were searched till September 2022. Risk of Bias was analyzed by using RoBDEMAT tool. Meta-analysis using Random Effects Model was performed for assessing the antimicrobial activity against *S. mutans, C. albicans* and *L. Acidophilus.*

**Results:**

A total of 11 studies were included the RoB analysis revealed sufficient reporting across all the domains and inconsistent reporting in only two of the domains. On qualitative analysis, a significant antimicrobial effect of TiO2 coating on orthodontic brackets against *Streptococcus mutans, Candida albicans* and *Lactobacillus acidophilus* was reported. The meta analysis revealed a significant overall antimicrobial effect with a high heterogeneity. (SMD: 3.5; p < 0.00001; i2 - 99.2%)

**Conclusion:**

An overall significant antimicrobial effect of TiO₂ coated brackets against *S. mutans, L. Acidophilus, C. Albicans* was noted but with a high heterogeneity. The subgroup analysis revealed a significant antimicrobial effect on *C albicans* with a low heterogeneity but it was limited by a publication bias. The included studies reported reduced surface roughness, minimal bacterial adhesion and less cytotoxic activity with TiO₂ coated brackets than uncoated brackets.

## Graphical abstract

**Figure:**
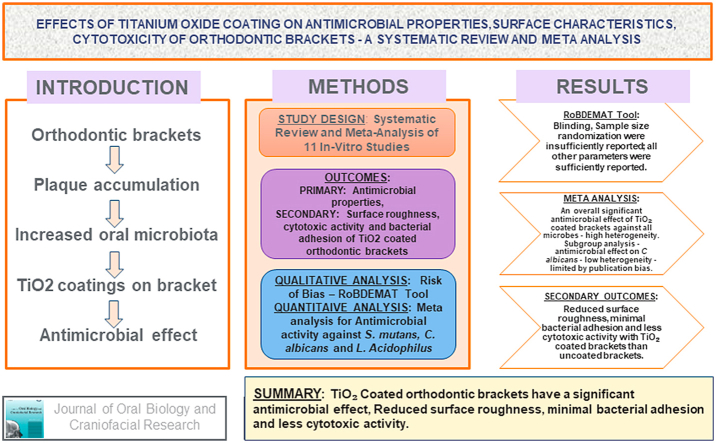

## 1 Introduction

The complex designs of orthodontic brackets make them an ideal niche for plaque accumulation and also hinders maintenance of oral hygiene. Bonding procedures itself can lead to plaque accumulation, demineralization and reduced pH of the plaque.^1^ Stainless steel brackets have a plaque retaining effect due to their higher critical surface tension and surface energy.^2^ *Streptococcus mutans* and *Lactobacillus Acidophilus* are two bacteria that are commonly associated with these changes in oral microbiota.^1^ White spot lesions are initial enamel demineralization areas seen around brackets in patients undergoing orthodontic treatment. White spot lesions have been known to occur as early as 1 month after placement of fixed orthodontic appliances.^3^ Various strategies have been suggested for management of white spot lesions like use of fluoride mouth rinses and dentifrices, use of probiotics and antibiotics, fluoride-releasing adhesive and professional scaling.^2^

Strategies not involving patient compliance in maintaining oral hygiene like coating of orthodontic brackets with semiconductor materials like titanium oxide, silver oxide, zinc oxide and many others have been tried recently. Titanium oxide (TiO₂) coating has been used in various industrial and environmental fields because of its chemically stable properties^4^, high photoactivity, stability, self-cleaning ability, biocompatibility^5^^,^^6^ and relatively low cost. When photocatalyzed, TiO₂ produces reactive oxygen species that promote bacterial membrane degradation, resulting in an antibacterial effect. TiO₂ exists in 3 crystalline forms: rutile, anatase, and brookite. The anatase and mixture (anatase + rutile) phases have effective antimicrobial activity against oral species.^7^ The photocatalytic activity of TiO₂ is best utilized by exposing it to ultraviolet radiation (UV-A). Although UV-A is beneficial, its clinical usage is not advisable due to potential hazards on human health.^8^ Hence, doping with metal and non-metal ions was introduced to reduce the optical gap to the UV-A spectrum of light.^9^ Nitrogen gained popularity among the non-metals on account of its enhanced optical properties.^10^ Various studies have been conducted on N-doped TiO₂ coating on orthodontic brackets.^11^ These studies stated there was increased antimicrobial activity when N-Doped TiO₂ coated brackets were exposed to visible light.^12^

Numerous other studies have shown that TiO₂ coated brackets and archwires have good antibacterial and anti-adherent properties.^13^^,^^14^ Short-term studies have shown that incorporating TiO₂ in orthodontic adhesives significantly improves antibacterial activity without compromising mechanical properties (e.g., shear bond strength).^15^ Previous research has studied the short term antimicrobial performance of orthodontic wires^16^ and brackets^17^ coated with photocatalytic TiO₂. However, long term clinical effectiveness and safety concerns of TiO₂ have not been explored.^18^ There is only one study^12^ that has evaluated the antibacterial effect of TiO₂ coated orthodontic brackets in a clinical setting, all others are in-vitro studies.

There have been no systematic reviews that report on the diverse implications of titanium oxide coating on orthodontic brackets. Since, there is no clinical trial on this topic, validation of the effects of coating TiO₂ on orthodontic brackets is required to employ them in orthodontic practice. This systematic review comprises of TiO₂ coated as well N-doped TiO₂ coated orthodontic brackets.

The objective of the current systematic review and meta-analysis is to critically analyze the available literature on the effects of titanium oxide coating on Orthodontic brackets.

## 2 Materials and methods

### 2.1 Protocol registration

The systematic review was prepared adhering to the reporting guidelines for Systematic reviews and Meta-Analysis in the PRISMA 2020 statement.^19^ The review has been registered with the PROSPERO database, International Prospective Register of Systematic Reviews with registration number - CRD42021251469.

### 2.2 Search strategy

To identify all peer-reviewed articles pertinent to the review's question, a systematic search of the medical literature published up to September 2022 was conducted. The following databases that were searched: Google Scholar, PubMed MEDLINE, Web of Science and SCOPUS. Grey literature sources like Open Grey and GreyNet International were also searched. The detailed search strategy is mentioned in Table 1. Similar keywords were used for the other databases as well. Reference lists of all the included articles were also manually searched for other relevant publications. Search was carried out by three authors (L.S, S.D, R.J). Only English literature was searched and the articles published until September 2022 were included. Duplicates were removed using the Endnote (version X9; Clarivate Analytics, Philadelphia, PA, USA) application.

**Table 1:** Search strategies.

PUB MED SEARCH	
Search Details	Results
(“orthodontic appliance*" [Title/Abstract]) OR (“orthodontic bracket*" [Title/Abstract])) OR ((“orthodontic appliances, fixed" [MeSH Terms]) OR (“orthodontic brackets" [MeSH Terms]))) AND ((((((“titanium dioxide" [Title/Abstract]) OR (“titanium oxide" [Title/Abstract])) OR (TiO2 [Title/Abstract])) OR (“titanium dioxide coated" [Title/Abstract])) OR (titanium oxide coated" [Title/Abstract])) OR (“TiO2 coated" [Title/Abstract]))) AND (((((((((((“antibacterial" [Title/Abstract]) OR (“antibacterial activity*" [Title/Abstract])) OR (“antibacterial effect*" [Title/Abstract])) OR (“antimicrobial" [Title/Abstract])) OR (“antimicrobial activity*" [Title/Abstract])) OR (“antimicrobial effect*" [Title/Abstract])) OR (“surface property*" [Title/Abstract])) OR (“cytotoxic activity*" [Title/Abstract])) OR (“cytotoxic effect*" [Title/Abstract])) OR (cytotoxic [Title/Abstract])) OR ((“bacterial adhesion" [MeSH Terms]) OR (“surface properties" [MeSH Terms])	**29**
“antibacterial" [Title/Abstract] OR “antibacterial activity*" [Title/Abstract] OR “antibacterial effect*" [Title/Abstract] OR “antimicrobial" [Title/Abstract] OR “antimicrobial activity*" [Title/Abstract] OR “antimicrobial effect*" [Title/Abstract] OR “surface property*" [Title/Abstract] OR “cytotoxic activity*" [Title/Abstract] OR “cytotoxic effect*" [Title/Abstract] OR “cytotoxic" [Title/Abstract] OR “bacterial adhesion" [MeSH Terms] OR “surface properties" [MeSH Terms]	5,93,640
“bacterial adhesion" [MeSH Terms] OR “surface properties" [MeSH Terms]	1,55,395
“antibacterial" [Title/Abstract] OR “antibacterial activity*" [Title/Abstract] OR “antibacterial effect*" [Title/Abstract] OR “antimicrobial" [Title/Abstract] OR “antimicrobial activity*" [Title/Abstract] OR “antimicrobial effect*" [Title/Abstract] OR “surface property*" [Title/Abstract] OR “cytotoxic activity*" [Title/Abstract] OR “cytotoxic effect*" [Title/Abstract] OR “cytotoxic" [Title/Abstract]	4,44,790
(((((“titanium dioxide" [Title/Abstract]) OR (“titanium oxide" [Title/Abstract])) OR (TiO2 [Title/Abstract])) OR (“titanium dioxide coated" [Title/Abstract])) OR (titanium oxide coated" [Title/Abstract])) OR (“TiO2 coated" [Title/Abstract])	33,781
“orthodontic appliance*" [Title/Abstract] OR “orthodontic bracket*" [Title/Abstract] OR “orthodontic appliances, fixed" [MeSH Terms] OR “orthodontic brackets" [MeSH Terms]	7863
“orthodontic appliances, fixed" [MeSH Terms] OR “orthodontic brackets" [MeSH Terms]	4763
“orthodontic appliance*" [Title/Abstract] OR “orthodontic bracket*" [Title/Abstract]	4746

## 3 Selection criteria

Eligibility criteria for including the studies in the review are mentioned in Table 2.

**Table 2:** PICO Analysis and eligibility criteria.

PICO	Inclusion criteria	Exclusion criteria
Population (P): Orthodontic brackets	Only ceramic and stainless-steel brackets	Wires, elastics, modules.
Intervention (I): Titanium oxide coating	Only Titanium oxide or N- doped TiO₂ coating	Other coatings like Ag, or nanoparticle coatings.
Comparison (C): uncoated orthodontic brackets	_	_
Outcomes (O): Primary: Antimicrobial activity Secondary: Surface roughness, Cytotoxic activity and bacterial adhesion.	Antimicrobial activity, surface roughness, cytotoxic activity and bacterial adhesion	Any other properties
Study Design	Only in-vitro studies	Randomized and non- randomized Control trials.

### 3.1 Screening and selection of studies

**Fig. 1:**
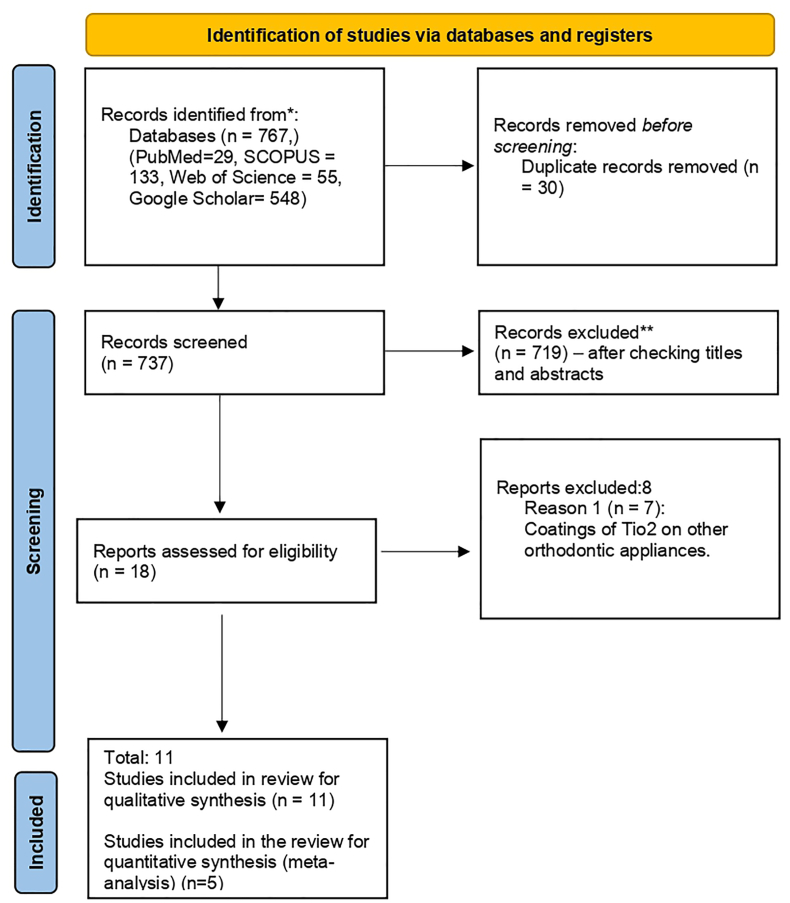
PRISMA flow diagram for including the studies in the review.

The review included all studies that met the selection criteria. The selection process of the studies for the review is depicted in Fig. 1. Three authors were involved in the study selection process, tabulation and bias assessment (LS, S.D, RJ). The intra-examiner agreement based on kappa statistics was 95%. Any disagreements were resolved by discussion with the fourth author (AB). The three authors (LS, S.D, RJ) extracted data from the included studies. Table 3 represents the study characteristics of the review which comprises all of the general information of the included studies.

**Table 3:** Characteristics table.

Author	Özyıldız, F. et al. 2009	AG Shah et al. 2011	Cao B et al. 2013	S. Cao et al. 2014	S. Cao et al. 2016	Fatani EJ et al. 2017	Ghasemi et al. 2017	Baby et al. 2017	Salehi et al. 2018	Math et al. 2021	Ameli et al., 2022
Samples	18	120	30	90	20	70	55	63	40	40	10
Groups (Brackets)	1:7 coated (A) 2: 5 uncoated 3: 6 coated in darkness (negative control)	1: Coated, 2: uncoated	1: N-doped TiO₂-xNy coated 2: Uncoated	1: N- Doped TiO₂ coated-annealed at 350°, 2: annealed at 450°, 3: annealed at 450, 4: uncoated (positive control), 5: uncoated (negative control)	1: Coated (1 layer) 2: coated (2 layers), 3: coated (3 layers), 4: Coated (4 layers), 5: coated (5 layers) 6: uncoated (positive control), 7: uncoated (negative control)	1: coated 2: uncoated	1: Coated (60 nm) 2: Coated (100 nm) 3: Uncoated	1:13 coated (A) 2: 13 coated (R) 3: 13 uncoated brackets (positive control)	1: N- Doped TiO₂ coated 2: Uncoated	1: 20 coated 2: 20 uncoated	1: Coated 2: Uncoated
Type of bracket	Ceramic	SS	SS	SS	Ceramic	SS	SS	SS	SS	SS	SS
Method of coating	Solgel dip	RF	RF	RF	Solgel dip	RF	PVD	RF	RF	RF	Solgel dip
Parameters	AA, SR	AA, BA	AA, SR. BA	AA, SR	AA, SR	AA, BA, BF, CA	AA, SR	AA, CA	AA	AA, BA	A.A
Micro-organisms	S.M	L.A	S.M, L.A, C.A, A. V	L.A, C. A	L.A, C. A	S.M, P. G	S.M	S.M	S.M	S.M	S.M
Methods	SEM, AFM, XRD, CFU at 16 h	CFU at 24 h	SEM, XRD, CFU at 24 h	CFU at 24 h SEM, XRD	CFU at 24 h, SEM, XRD	SEM, (PBS) for adhesion assays, Optical density for AA	SEM, AFM, INSTRON, CFU at 3, 6, 24, and 48 h	CFU at 24 h	CFU at 24 h, 30, 60 and 90 days, XRD	CFU 24 h	CFU at 24 h, 1 wk, 1 and 3 months

A: Anatase phase; R: Rutile phase; SS: Stainless steel brackets; RF: Radiofrequency magnetron sputtering method; PVD: Physical vapor deposition method; AA: Antimicrobial activity; SR: Surface roughness; BA: Bacterial Adhesion; CA: Cytotoxic activity; *S.M: Streptococcus mutans; L.A: Lactobacillus Acidophilus; C.A: Candida albicans; A.V: Actinomycoses viscosus; P.gingivalis;* CFU: Colony forming units; SEM: Scanning Electron Microscopy; AFM: Atomic Force Microscopy; XRD: X-Ray Power Diffraction; PBS: Phosphate buffered saline, h-hours.

### 3.2 Qualitative assessment

For the qualitative assessment, a recently developed RoBDEMAT risk of bias (RoB) assessment tool was employed .^20^ The included studies were assessed based on the following sources of bias: Bias in planning (control group, sample randomization, and sample size rationale); bias in sample preparation (standardization of samples, use of identical experimental conditions); bias in outcome assessment (blinding and standardization of testing procedures); bias in data treatment and outcome reporting (statistical analysis and reporting of study outcomes). A table summarizing the RoB results is depicted in Table 4. Each signaling question was reported as “sufficiently reported”, “insufficiently reported”, “not reported”, or “not applicable”. Authors of the RoB tool mention that an overall summary RoB score is not to be produced, since it was a simple checklist.

**Table 4:** Assessment of the risk of bias in the included studies.

Author		Özyıldız, F. et al., 2009	AG Shah et al., 201	Cao B et al., 2013	S. Cao et al. 2014	S. Cao et al. 2016	Fatani EJ et al., 2017	Ghasemi et al., 2017	Baby et al., 2017	Salehi et al. 2018	Math et al., 2021	Ameli et al., 2022
**(D1)** Bias in planning	**1.1** Control group	SR	SR	SR	SR	SR	SR	SR	SR	SR	SR	SR
	**1.2** Randomization of samples	NR	NR	NR	NR	NR	NR	NR	NR	NR	NR	SR
	**1.3** Sample size rationale and reporting	IR	IR	IR	IR	IR	IR	IR	SR	IR	IR	IR
**(D2)** Bias in sample/specimen preparation	**2.1** Standardization of samples and materials	SR	SR	SR	SR	SR	SR	SR	SR	SR	SR	SR
	**2.2** Identical experimental conditions	SR	SR	SR	SR	SR	SR	SR	SR	SR	SR	SR
**(D3)** Bias in outcome assessment	**3.1** Adequate and standardized testing procedures and outcomes	SR	SR	SR	SR	SR	SR	SR	SR	SR	SR	SR
	**3.2** Blinding of the test operator	NR	NR	NR	NR	NR	NR	NR	NR	NR	NR	NR
**(D4)** Bias in data treatment and outcome reporting	**4.1** Statistical analysis	SR	SR	SR	NR	SR	SR	SR	SR	SR	SR	SR
	**4.2** Reporting study outcomes	SR	SR	SR	SR	SR	SR	SR	SR	SR	SR	SR

*Judgment scoring:* “sufficiently reported” (SR); “insufficiently reported” (IR); “not reported” (NR); “adequate”; “not adequate”.

### 3.3 Quantitative assessment of the included studies

**Fig. 2:**
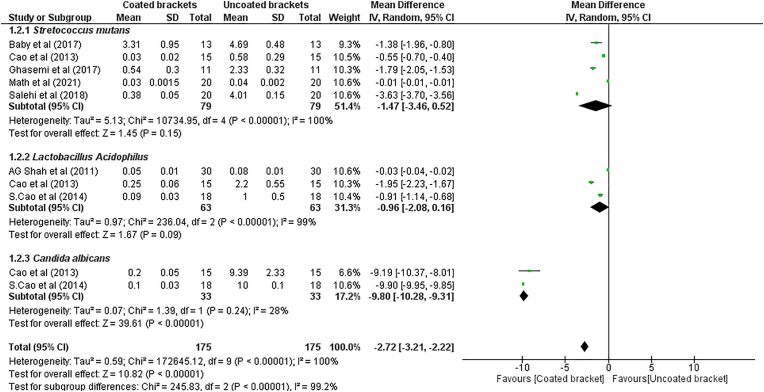
Random effects model for quantitative assessment of antimicrobial activity of TiO₂ coated Orthodontic brackets against 1.2.1) S. mutans (MD -1.47, 95% CI -3.46 to 0.52); 1.2.2) L. Acidophilus (MD -0.96, 95% CI -2.08 to 0.16); 1.2.3) C. Albicans (MD -9.80, 95% CI -10.28 to −9.31).

Meta-analysis of the primary outcomes was performed using Cochrane review manager software (Revman version 5.4). The overall effects were calculated using a random effects model (DerSimonian-Laird random effects pooling method). A subgroup meta-analysis with pooled mean difference (Fig. 2) was done for the antimicrobial effect of TiO₂ coated brackets against *S. mutans, L. Acidophilus, C. albicans* at 24h incubation period. The antimicrobial effect was expressed in the form of mean CFU/ml. All the articles had different dilutions which were converted to 10⁵ CFU/ml to obtain homogenous data to perform meta-analysis. To assess the presence of publication bias a funnel plot was used.

## 4 Results

### 4.1 Study selection

The electronic search identified a total of 767 articles. After removing the duplicates, 737 studies were further screened. After screening of the titles and abstracts, only 18 studies were selected. On further screening of the studies for eligibility criteria 7 studies were excluded. 6 articles were excluded as the coating of TiO2 was either on other orthodontic appliances or incorporated in bonding agents and adhesives. One of the recent studies by Monica et al. was excluded as it was a Randomized control trial and used a different method as compared to other included studies for measuring antibacterial activity.^12^ Finally, 11 studies were relevant and were included for qualitative analysis. 5 studies were selected for quantitative analysis (Fig. 1).

### 4.2 Study characteristics

Table 3 provides a summary of the characteristics of the eleven studies that were included. Antimicrobial activity was the primary outcome evaluated by all of the included studies. The antimicrobial effect of the coated brackets against *Streptococcus mutans; Lactobacillus Acidophilus; Candida albicans; Actinomycoses viscosus* and *P. gingivalis* was assessed as the number of Colony Forming Units (CFU/ml ✕ 10⁵). The secondary outcomes assessed were surface roughness, bacterial adhesion and cytotoxic activity of the orthodontic brackets.

### 4.3 Risk of bias within included studies

Risk of bias assessment was evaluated using the RoBDEMAT tool .^20^ For the domain bias in planning (D1), all the included studies reported a control group. None of the included studies reported on randomization and allocation of the samples except for the study by Ameli et al. who had reported it sufficiently .^21^ Sample size calculation and reporting was carried out in only one of the studies .^22^ The remaining studies either provided insufficient information or did not provide information on sample size calculation. Overall, for D1, a greater inconsistency was noted. For the domain bias in sample/specimen preparation (D2), all the studies had sufficiently reported for sample standardization and for identical experimental conditions. For the domain, bias in outcome assessment (D3), adequate and standardized testing procedures and outcomes were sufficiently reported in all studies. Operator blinding was not reported in any of the included studies. For the domain, bias in data treatment and outcome reporting (D4) all the studies reported sufficiently, except a study by S.Cao et al. failed to quantitatively assess their data. ^23^

### 4.4 Summary of findings

Tables 5 and 6 give the results of the primary and secondary outcomes respectively. 8 studies evaluated the antimicrobial effect of titanium oxide coated brackets against *S.mutans* and all reported lesser CFUs/ml of *S.mutans* when compared with uncoated brackets indicating a good antimicrobial effect.^11^^,^^22^^,^^24^, ^25^, ^26^^,^^17^^,^^21^ 4 of the included studies evaluated the antimicrobial activity against *L.acidophilus*^8^^,^^23^^,^^27^^,^^28^, 4 studies against *C.albicans*^23^^,^^24^^,^^8^^,^^28^, 1 against *A.viscosus*^8^ and 1 against *P.gingivalis*.^25^ All of the studies reported that TiO₂ coated brackets had a better antimicrobial effect than uncoated brackets, except against *A. viscosus*.

**Table 5:** Results of primary outcome.

Author	Antimicrobial activity: (Cfu/ml) × 10^5^					P value	Conclusion
Özyıldız, F. et al., 2009 Incubation period: 16 h	*S. Mutans*		*C. Albicans*			(p < 0.05)	TiO₂ coated brackets illuminated with UV- A reduced 98% of S.M and 93% of C.A colonies.
	Groups						
	1: 0.2 +/- 0.05		1: 0.00001				
	2: 6 +/- 1		2: 0.00011 +/- 0.00003				
	3: 7 +/-2		3: 0.0012 +/- 0.0003				
AG Shah et al. 2011 Incubation period: 24 h	*L.Acidophilus*					(p < 0.01)	TiO₂ coating had bactericidal effect against L.A.
	1: 0.052 +/- 0.013						
	2:0.08 +/- 0.013						
Cao B et al., 2013 Incubation period: 24 h	*S. Mutans*	*C.Albicans*	*L.Acidophilus*	*A. viscosus*		(p < 0.001)	TiO₂ coated brackets have high AA against S.M, C.A, L.A, weak prevention for A.V.
	1: 0.03+/- 0.02	1: 0.20 +/- 0.05	1: 0.25+/- 0.06	1: 0.08 +/- 0.02			
	2: 0.58 +/- 0.29	2: 9.39 +/- 2.33	2: 2.2 +/- 0.55	2: 0.28 +/- 0.07			
S. Cao et al. 2014 Incubation period: 24 h	*L.Acidophilus*		*C.Albicans*			–	TiO₂ films annealed at 450 °C had best AA against L.A, C.A.
	1: 0.07 +/- 0.02		1:0.08+/- 0.02				
	2: 0.09 +/- 0.02		2: 0.1 +/- 0.03				
	3: 0.05+/- 0.01		3: 0.06 +/- 0.02				
	4: 1 +/- 0.25		4: 10 +/- 0				
	5: 0.09 +/- 0.03		5: 0.1 +/- 0.02				
S. Cao et al. 2016 Incubation period: 24 h	*L.Acidophilus*		*C.Albicans*			(p < 0.05)	TiO₂ films with 5-layer coating annealed at 700 °C had greatest AA against L.A, C.A.
	1: 0.71 +/- 0.2		1: 0.78 +/- 0.2				
	2: 0.54 +/- 0.14		2: 0.64 +/- 0.16				
	3: 0.31+/- 0.08		3: 0.43 +/- 0.10				
	4: 0.19+/- 0.04		4: 0.29 +/-0.07				
	5: 0.08 +/- 0.02		5: 0.18+/- 0.04				
	6: 0.92 +/- 0.23		6: 0.86 +/- 0.21				
	7:1.00 +/-0.25		7: 1.00 +/- 0.25				
Fatani EJ et al., 2017	*S. Mutans*		*P. gingivalis*			(p < 0.05)	TiO₂ coated brackets had good AA against S.M, P.G.
	Groups:						
	1: 0.39 +/- 0.02		1: 0.35 +/- 0.02				
	2: 0.76 +/- 0.05		2: 0.51 +/- 0.02				
Ghasemi et al., 2017 Incubation period: 3,6,24,48 h	*S. Mutans*					(p < 0.05)	TiO₂ coated groups showed significant reduction in S.M colony counts.
	Groups:						
	1 (60 nm):		2 (100 nm):				
	3 h: 1.74+/- 0.14		3 h: 1.68 +/- 0.14				
	6 h: 1.19+/- 0.29		6 h: 0.97 +/- 0.29				
	24 h: 0.82 +/- 0.32		24 h: 0.54 +/- 0.32				
	48 h: 0.66 +/- 0.22		48 h: 0.42 +/- 0.22				
	3:						
	3 h: 1.82 +/- 0.14						
	6 h: 2.07 +/-0.29						
	24 h:2.33 +/- 0.32						
	48 h: 12.59 +/- 0.22						
Baby et al., 2017 Incubation period: 24 h	*S. Mutans*					(p < 0.5)	R phase of TiO₂ had better AA than A phase.
	Groups:						
	1(A) 3.31 +/- 0.95						
	1(R): 2.3 +/- 0.75						
	2: 4.69 +/- 0.48						
Salehi et al. , 2018, Incubation period: 24 h, 30,60,90 days	*S. Mutans*					(p < 0.001)	Strongest AA against S.M over a period of 90 days.
	Groups	24 h	30 days	60 days	90 days		
	1	4.01+/- 0.15	4.02 +/- 0.14	4.01 +/-0.15	4.01+/- 0.14		
	2	0.38+/- 0.05	0.38 +/- 0.05	0.38+/- 0.054	0.3 +/- 0.05		
	Mean: Group 1: 4.01 +/- 0.52,		Group 2: 0.04 +/- 0.02				
Math et al., 2021 Incubation period: 24 h.	*S. Mutans*					(p = 0.000414)	TiO₂ coated groups showed significant reduction in S.M colony counts.
	Groups						
	1: 0.031 +/- 0.0015						
	2: 0.038+/- 0.0019						
Ameli et al., Incubation period: 24 h.1 wk,1 and 3 months	*S. Mutans*					P < 0.05	TiO₂ coated groups showed increased S.M colony counts after 1 wk.
	Groups	24 h	1 wk	1mnth	3 mnth		
	1	0.475 +/- 0.09	4.72 +/- 0.19	5.54 +/- 0.47	8.58 +/- 1.15		
	2	Not mentioned (higher colony count of S.M than the coated group at all points of time)					

(A: Anatase phase; R: Rutile phase; AA: Antimicrobial activity; *S.M: Streptococcus mutans; L.A: Lactobacillus Acidophilus; C.A: Candida albicans; A.V: Actinomycoses viscosus; P.gingivalis;* h-hours.

**Table 6:** Results of secondary outcome.

Author	Surface roughness	Adhesion assay	Biofilm formation	Cytotoxic activity
F. Özyildilz et al., 2009	Coated brackets have a smoother surface.	–	–	–
AG Shah et al., 2011		Greater bacterial adhesion to uncoated brackets	–	–
Cao et al., 2013	–	Greater bacterial adhesion to uncoated brackets.	–	–
S. Cao et al., 2014	Coated brackets have a smoother surface.	–	–	–
Fatani et al., 2017	–	Greater bacterial adhesion on uncoated brackets.	Lesser on coated brackets.	Coated brackets have lesser cytotoxicity.
Ghasemi et al., 2017	Coated brackets have a smoother surface.	–	–	–
Baby et al., 2017	–	–	–	Anatase phase of TiO₂ had lesser cytotoxicity than rutile phase of TiO₂ and control group.
Math et al., 2021	–	Greater bacterial adhesion on uncoated brackets.	–	

5 studies^23^^,^^8^^,^^11^^,^^24^^,^^28^ summarized that the anatase phase of TiO₂ had greater antimicrobial activity but one study^22^ stated that the rutile phase had a higher antimicrobial effect against *S.mutans*.

3 of the studies^23^^,^^11^^,^^24^ used N-doped TiO₂ thin film coatings on the brackets. 3 studies reported that Coated brackets had a smoother surface than the uncoated ones.^23^^,^^17^^,^^24^ 4 studies reported that bacterial adherence^8^^,^^25^^,^^26^^,^^27^ was lesser on coated brackets. 2 studies reported lesser cytotoxic effect with coated brackets.^25^^,^^22^ Baby et al. reported that the anatase phase has lesser cytotoxicity than the rutile phase of TiO₂^22^ .

### 4.5 Results of quantitative analysis

The meta-analysis of the 5 included studies reported the antimicrobial effect of the orthodontic brackets coated with TiO2. The study conducted by Ameli et al.^21^ was excluded from the meta-analysis as the data for the control group was not tabulated by the authors.

The overall heterogeneity across the studies was found to be high (I2 = 99.2%). Thus, a random effects model was used to quantitatively assess the antimicrobial effect. Fig. 2 shows no significant pooled mean difference between coated and uncoated brackets (SMD = −1.47; p value = 0.15; 95% CI = −3.46 to 0.52) for number of CFUs of *S. mutans* and *for L. acidophilus* (SMD = −0.93; p value = 0.09; 95% CI = −2.08 to 0.16). However, a significant pooled mean difference (SMD = −9.8; p value = 0.00001; 95% CI = −10.28 TO -9.31) was noted for *C. albicans* favoring coated brackets. The pooled CFUs of *C. albicans* was found to be less for coated brackets compared to uncoated brackets. An overall significant antimicrobial effect was reported (SMD: −3.5; p value < 0.00001; CI = −6.86 to 0.36). There is a substantial unexplained heterogeneity between the included studies within each of the subgroups. Therefore, the validity of the total effect estimate for each subgroup is uncertain.

### 4.6 Publication bias

**Fig. 3:**
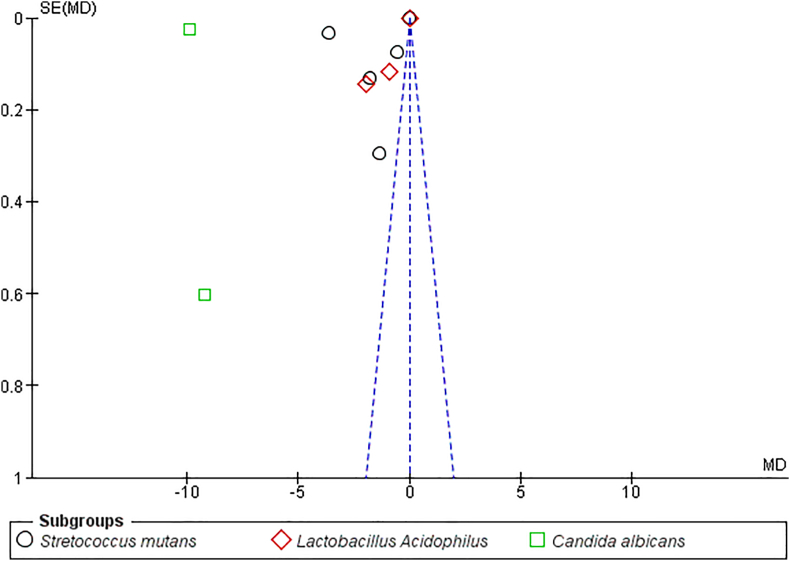
Funnel plot for publication bias.

A funnel plot represents the presence of publication bias in the included studies due to increased standard error (0.6) in one of the included studies^8^ for *C. albicans*. This increased standard error will affect the certainty of evidence of the review [Fig. 3].

## 5 Discussion

The systematic review aimed at critically analyzing the available literature on the effects of TiO₂ coating on the antimicrobial activity, surface characteristics and cytotoxicity of orthodontic brackets. This is a first systematic review assessing the effects of TiO₂ coating on orthodontic brackets. Eight studies investigated the antimicrobial effect of TiO₂ coated brackets against *S mutans* and found a significant antimicrobial effect but when subjected to meta-analysis no significant antimicrobial effect was noted (p value = 0.15). Three studies stated that TiO2 coated brackets had a good antimicrobial effect against *L. Acidophilus* but on quantitative analysis no such effect was noted (p value = 0.09). On the contrary, three studies which reported good antimicrobial effects of TiO₂ coated brackets on *C. albicans*, when subjected to quantitative analysis showed statistically significant antimicrobial effects. The results of the quantitative analysis revealed an overall significant reduction in the bacterial count for brackets coated with TiO₂ as reported by the included studies. Brackets coated with TiO₂ had a lesser surface roughness, less bacterial adhesion and less cytotoxic activity compared to uncoated brackets but a quantitative analysis could not be performed for these parameters as the results of these parameters were not represented quantitatively. Overall, the meta-analysis reported a high heterogeneity (I2 = 99.2%). The subgroups also reported a high heterogeneity except *C. Albicans* which had a low heterogeneity (I2 = 28%).

The risk of bias was assessed using the RoBDEMAT tool, adapted from the study by Delgado et al.^20^ This RoB tool does not give a subjective cumulative score but is merely a nine-step checklist to assess the quality of evidence of the included studies. Most studies either reported insufficiently or did not report on bias in planning and allocation, sample size calculation, and randomization. Operator blinding was not reported in any study. Hence, these shortcomings contributed to greater inconsistency. Bias in outcome assessment (D3), consisting of standardized testing procedures and outcomes, was reported sufficiently in most studies. Biases in data treatment and outcome reporting (D4), which included statistical analysis and reporting study outcomes were sufficiently reported across all studies except in one of the studies. RoBDEMAT tool was used as it is a validated tool for systematic reviews and meta-analysis based on in-vitro studies 20.

Presence of publication bias was noted in this review as there was a high standard error (0.6) in one of the included studies^8^ (Fig. 3).

### 5.1 Antimicrobial activity

All of the included studies have assessed the antimicrobial activity and reported that coated brackets have significantly higher antimicrobial activity than uncoated brackets. The Random effects model revealed that TiO₂ coated brackets had an overall significant higher antimicrobial activity against the microbes studied (p < 0.00001). A random effects model was used for quantitative analysis of the results as there were methodological inconsistencies across the studies and these could have contributed to the high heterogeneity [*S. mutans* (i2- 100%) *and L. acidophilus* (i2- 99%)] across subgroups as well as to the overall high heterogeneity (i2- 99.2%). After visual examination of the forest plot, we attempted sensitivity analysis to eliminate the studies showing skewed results which may have contributed to high heterogeneity, however even after excluding those studies, the heterogeneity remained substantially high. Previous systematic reviews of in vitro studies have reported moderate to high heterogeneity (Dumbryte et al. (i2- 97%).^29^ and Pourhajibagher et al. (i2 - 77.3%)^30^ Israel et al.^31^ stated that despite the existence of statistical tests for heterogeneity, there are no officially recognized guidelines for when a meta-analysis should not be carried out as a result of statistical heterogeneity. There was a significant decrease in *C. Albicans CFUs* with a low heterogeneity (i2-28%). However, the results were contributed by merely 3 studies and the funnel plot analysis revealed a high standard error^8^ contributing to a possible publication bias. Hence, the certainty of the evidence is questionable. Cao et al.^8^ revealed weak antimicrobial activity against *A. viscosus.* Fatani et al. assessed the antimicrobial activity of the coated brackets against *P. gingivalis* and showed a moderate antimicrobial potential.^25^

The antimicrobial effect of TiO₂ coatings was due to release of hydroxyl ions. TiO₂ exhibited this property either by UV light illumination^8^^,^^17^^,^^23^^,^^24^^,^^26^^,^^27^ or by N-doping in visible light spectrum.^8^^,^^11^^,^^28^ Therefore, visible regions have more clinical acceptability.^32^ Most studies claim the annealing temperature of 450° at 2 h results in the formation of anatase phase of TiO₂ which has better antimicrobial activity than the other phases. F. Özyildilz et al.^24^ and S. Cao et al.^28^ showed strong antimicrobial potential at 500 degrees celsius and 700 degrees celsius respectively.

There is only one recent randomized control trial carried out on the antimicrobial effect of N-doped TiO₂ coated orthodontic brackets.^12^ The results of this study reveals decreased concentrations of *S. mutans* 30 days after placement of orthodontic appliances as compared to control group. The results of a previous systematic review conducted by Siva S et al. on nanocoated orthodontic brackets and their results are consistent with the results of our systematic review but their review was not specific for TiO₂ and included other coatings like nanosilver, silver-platinum, zinc oxide and copper oxide of orthodontic brackets. Previous studies have evaluated the antimicrobial effect of other coatings on orthodontic brackets. An in-vivo study by G. Metin-Gürsoy et al. evaluated the antimicrobial effect of nanosilver (Ag) coated brackets against *S. mutans* and showed statistically significant decrease in the CFU/ml as compared to uncoated brackets.^33^ Similar antibacterial effects of nanosilver coated brackets were exhibited in an in-vitro study by Arash et al.^34^ Another in-vitro study assessed the antimicrobial effect of silver-platinum coating on orthodontic brackets and elicited an acceptable antimicrobial effect against *S. mutans*.^35^ Brackets coated with CuO, ZnO and CuO-ZnO had higher antibacterial properties than their uncoated counterparts.^36^ A study by Zhang et al. stated a higher antibacterial effect of nano Ag-Tio2 coated orthodontic brackets against various organisms.^37^ The results of all these aforementioned studies are in favor of coated brackets and the results of our systematic review and meta analysis highlights the greater antimicrobial effect of TiO₂ coated orthodontic brackets specifically as compared to the uncoated conventional brackets.

### 5.2 Surface roughness

Three studies^23^^,^^17^^,^^24^ concluded that the surface roughness of the coated brackets was much lesser than the uncoated brackets. An Experimental study by Zhang et al.^37^ stated that nano - TiO₂, Ag-Tio2 coatings had smaller particle size and smoother surfaces as suggested by Scanning electron microscopic analysis. Rough surfaces have an affinity towards bacterial adhesion.^38^ Reduction in the surface roughness by TiO₂ coatings lays a foundation for lesser bacterial adherence, lesser plaque accumulation and reduces the prevalence of White spot lesions and periodontal diseases.

### 5.3 Bacterial adhesion

Four^26^^,^^8^^,^^25^^,^^27^ of the included studies showed significant reduction in bacterial adhesion. Bacterial adhesion was assessed by counting microbial colonies^8^ or by weighing brackets before and after cultures.^27^^,^^26^ Lesser biofilm formation of *S. mutans and P. gingivalis* was noted on coated brackets when compared to uncoated brackets.^25^ These effects were observed because of the release of hydroxyl ions from TiO2 that react with surface molecules of bacteria causing surface decomposition and the formation of a fragile wall.

### 5.4 Cytotoxic activity

Even though TiO₂ has been proved to be a biocompatible material, the corrosion products from the surface of the bracket due to the mechanical stress and wear might contribute to some amount of toxicity.^39^ Two studies in our systematic review have reported the cytotoxic effects of TiO₂. TiO₂ coated brackets had less cytotoxicity as quoted by Fatani et al.^25^ The Anatase phase of TiO₂ was only fractionally cytotoxic as compared to uncoated brackets with no statistically significant difference between the two, whereas the rutile phase had greater cytotoxicity as compared to the uncoated brackets.^22^ Baby et al. stated that the rutile phase of TiO₂ had greater antimicrobial activity than the anatase phase. They recommended the use of anatase phase for coating on brackets owing to its significant bacterial effect and less cytotoxicity as compared to the rutile phase.

Nano TiO₂ and nano Ag-TiO₂ coatings on orthodontic brackets possess good biocompatibility according to a study by Zhang *et* al.^37^ Other coatings like Ag also have been proven to exhibit lower cytotoxic activity.^35^

There are reports in the literature on the effects of TiO₂ coatings on other orthodontic appliances. Venkatesan et al. reported a significant antibacterial effect of TiO₂ and a reduced surface roughness, but only for a period of 1 month. They questioned the long-term effects of TiO₂ due to its wear over a period of time.^40^ Wires coated with TiO₂ had lesser bacterial adhesion and good antibacterial properties against *S. mutans* and *P. gingivalis*.^41^ A study on TiO₂ coated wires stated no cytotoxic effect of TiO₂ coatings.^42^

The results of the meta-analysis records the antimicrobial effect of TiO₂ coated brackets at only 24 h. Few studies have reported the antimicrobial effect up to 90 days. This data could not be included in the meta-analysis as it would add to the heterogeneity of the results. Also, this systematic review and meta-analysis gives a combined result on the antimicrobial effect of TiO₂ coated as well as N-doped TiO₂ coated orthodontic brackets. Future studies need to be conducted to find out the difference in the properties of TiO₂ coated as well as N-doped TiO₂ coated orthodontic brackets.

Studies included in the review presented various methodological inconsistencies like varying incubation periods, different culture mediums and the type and thickness of TiO₂ coatings. Some of the studies had a small sample size and the majority of them did not report on sample size calculation. Furthermore, no study assessed the antimicrobial effect on a multispecies biofilm as formed on a saliva pellicle that closely resembled the oral cavity microbiota. Standardization of methods would be useful for future research. High heterogeneity was also reported in the random effect model for subgroups *S. mutans* and *L. acidophilus.* The data on the toxicologic effects and surface characteristics of TiO₂ presented in this systematic review is limited. There is a dire need to conduct more studies on these aspects. This was a systematic review of in-vitro studies; additional animal studies and human trials are needed to validate the presented results.

### 5.5 Clinical relevance

Bacterial colonization of orthodontic brackets in the oral cavity can lead to demineralization of the surrounding enamel. TiO₂ coated brackets offer antimicrobial effect, smoother surfaces and less bacterial adhesion hence they can be used in routine orthodontic practice to reduce plaque accumulation and white spot lesions.

## 6 Conclusions

Within the limitations of the systematic review, the following conclusions could be deduced.

· An overall significant antimicrobial effect of TiO₂ coated brackets against *S. Mutans, L. Acidophilus, C. Albicans* was noted but with a high heterogeneity, hence caution should be exercised before considering clinical use.
· The subgroup analysis revealed a significant antimicrobial effect on *C Albicans* with a low heterogeneity but it was limited by a publication bias in one of the included studies.
· The included studies reported reduced surface roughness, minimal bacterial adhesion and less cytotoxic activity with TiO₂ coated brackets than uncoated brackets.

## Funding sources

This research did not receive any specific grant from funding agencies in the public, commercial, or not-for-profit sector.

## Author statement

Lichi Solanki, S.P: Conceptualization, Data curation, Formal analysis, Methodology, Software, Supervision, Roles/Writing - original draft, Writing - review & editing. Saravana Dinesh: Conceptualization, Data curation, Formal analysis, Methodology, Validation, Visualization, Writing - review & editing. Ravindra Kumar Jain: Conceptualization, Data curation, Formal analysis, Methodology, Supervision, Validation, Visualization, Writing - review & editing. Arthi Balasubramaniam: Software, Validation.

## List of abbreviations

A: Anatase phase
R: Rutile phase
SS: Stainless steel brackets
RF: Radiofrequency magnetron sputtering method
PVD: Physical vapor deposition method
AA: Antimicrobial activity
SR: Surface roughness
BA: Bacterial Adhesion
CA: Cytotoxic activity
*S.M*: *Streptococcus mutans*
*L.A*: *Lactobacillus Acidophilus*
*C.A*: *Candida albicans*
*A.V*: *Actinomycoses viscosus*
*P*: *gingivalis*
CFU: Colony forming units
SEM: Scanning Electron Microscopy
AFM: Atomic Force Microscopy
XRD: X-Ray Power Diffraction
PBS: Phosphate buffered saline, h-h
TiO₂: Titanium oxide.
